# A complex culturally targeted intervention to reduce Hispanic disparities in living kidney donor transplantation: an effectiveness-implementation hybrid study protocol

**DOI:** 10.1186/s12913-018-3151-5

**Published:** 2018-05-16

**Authors:** Elisa J. Gordon, Jungwha Lee, Raymond H. Kang, Juan Carlos Caicedo, Jane L. Holl, Daniela P. Ladner, Michelle D. Shumate

**Affiliations:** 1 Department of Surgery-Division of Transplantation, Center for Healthcare Studies, Center for Bioethics and Medical Humanities, Northwestern University Feinberg School of Medicine, 633 N. St. Clair, 20th FL, Chicago, IL 60611 USA; 2 Preventive Medicine and Biostatistics Collaboration Center, Northwestern University Feinberg School of Medicine, 680 N. Lake Shore Drive, Suite 1400, Chicago, IL 60611 USA; 3 Center for Healthcare Studies, Northwestern University Transplant Outcomes Research Collaborative (NUTORC), Northwestern University Feinberg School of Medicine, 633 N. St. Clair, 20th FL, Chicago, IL 60611 USA; 4 Department of Surgery-Division of Transplantation, Northwestern University Feinberg School of Medicine, 676 N. St. Clair, 19th FL, Chicago, IL 60611 USA; 5 Center for Healthcare Studies, Northwestern University Feinberg School of Medicine, 633 N. St. Clair, 20th FL, Chicago, IL 60611 USA; 6 Department of Communication Studies, Northwestern University, 240 Campus Drive, Rm 2-118, Evanston, IL 60208 USA

**Keywords:** End-stage kidney disease, Health disparities, Equity, Organizational-level intervention, Hybrid study, Implementation science, Complex interventions

## Abstract

**Background:**

The shortage of organs for kidney transplantation for patients with end-stage renal disease (ESRD) is magnified in Hispanics/Latin Americans in the United States. Living donor kidney transplantation (LDKT) is the treatment of choice for ESRD. However, compared to their representation on the transplant waitlist, fewer Hispanics receive a LDKT than non-Hispanic whites. Barriers to LDKT for Hispanics include: lack of knowledge, cultural concerns, and language barriers. Few interventions have been designed to reduce LDKT disparities. This study aims to reduce Hispanic disparities in LDKT through a culturally targeted intervention.

**Methods/Design:**

Using a prospective effectiveness-implementation hybrid design involving pre-post intervention evaluation with matched controls, we will implement a complex culturally targeted intervention at two transplant centers in Dallas, TX and Phoenix, AZ. The goal of the study is to evaluate the effect of Northwestern Medicine’s® Hispanic Kidney Transplant Program’s (HKTP) key culturally targeted components (outreach, communication, education) on Hispanic LDKT rates over five years. The main hypothesis is that exposure to the HKTP will reduce disparities by increasing the ratio of Hispanic to non-Hispanic white LDKTs and the number of Hispanic LDKTs. We will also examine other process and outcome measures including: dialysis patient outreach, education session attendance, marketing efforts, Hispanic patients added to the waitlist, Hispanic potential donors per potential recipient, and satisfaction with culturally competent care. We will use mixed methods based on the Promoting Action on Research Implementation in Health Services (revised PARIHS) and the Consolidated Framework for Implementation Research (CFIR) frameworks to formatively evaluate the fidelity and innovative adaptations to HKTP’s components at both study sites, to identify moderating factors that most affect implementation fidelity, and to identify adaptations that positively and negatively affect outcomes for patients.

**Discussion:**

Our study will provide new knowledge about implementing culturally targeted interventions and their impact on reducing health disparities. Moreover, the study of a complex organizational-level intervention’s implementation over five years is rare in implementation science; as such, this study is poised to contribute new knowledge to the factors influencing how organizational-level interventions are sustained over time.

**Trial registration:**

(ClinicalTrials.gov registration # NCT03276390, date of registration: 9–7-17, retrospectively registered)

## Background

More than 600,000 patients in the U.S. have end-stage kidney disease (ESRD) for which dialysis or transplantation are the only life-saving options [1]. Disproportionately more ESRD patients are Hispanics/Latin Americans (“Hispanics”) than non-Hispanic whites [1], which contributes to the greater demand for kidney transplantation. Thus, while the shortage of kidneys for transplantation adversely affects all patients with ESRD, the shortage is accentuated for Hispanics [2, 3]. In 2016, disproportionately fewer waitlisted Hispanics than non-Hispanic whites received kidney transplants: 17.8% versus 25.1% [4], despite having a disproportionately higher prevalence of ESRD [1, 5–8]. Living donor kidney transplant (LDKT) is the optimal treatment for ESRD as it engenders longer patient and graft survival, shorter waiting time, and better quality of life than deceased donor kidney transplantation [9–12]. Further, transplantation is more cost-effective than dialysis [1]. However, in 2016, disproportionately fewer waitlisted Hispanics than non-Hispanic whites received a LDKT: 4.6% versus 10.5% [13]. This disparity will likely worsen without a change in current medical practice as Hispanics are the largest and fastest growing ethnic minority group in the United States (U.S.) [14, 15].

Barriers to LDKT specific to Hispanics are due to: misconceptions about living kidney donation (LKD), fears about not being able to have children, fears about shortening the donor’s life expectancy [16], lack of educational materials about transplantation in Spanish [17, 18], concerns over legal documentation, and preferences for “high context communication” [19] or education provided through face-to-face and group settings [16, 18, 20]. Hispanics also maintain concerns about LDKT that other groups have expressed, including: lack of knowledge about LKD [8, 18, 21–24], fear of surgery [16, 21, 23], financial concerns [18, 23, 25, 26], distrust of healthcare providers [19, 21, 23], recipient reluctance to request donation [23, 25, 27], and patients’ refusal of living donation out of concern for donor well-being [23].

Culturally competent or targeted interventions have contributed to the reduction in health disparities [28–30]. Cultural competency pertains to: “A set of values, principles, behaviors, attitudes, policies, and structures that enable organizations and individuals to work effectively in cross-cultural situations.” [31]. A feature of cultural competency is cultural congruence, which refers to: “those cognitively based assistive, supportive, facilitative, or enabling acts or decisions that are tailor made to fit with individual, group, or institutional cultural values, beliefs, and lifeways in order to provide or support meaningful, beneficial, and satisfying health care or well-being services” [32] and occurs when patients and providers share the same ethnic background, cultural beliefs, and values. Both concepts rely on the concept of “culture,” defined as: “a generalized, coherent context of shared symbols and meanings that persons dynamically create and recreate for themselves in the process of social interaction… culture is something people come to take for granted...” [33] Linguistic congruence entails: “The capacity of an organization and its personnel to communicate effectively, and convey information in a manner that is easily understood by diverse audiences including persons of limited English proficiency, those who have low literacy skills or are not literate…” such as through bilingual/bicultural or multilingual/multicultural staff [34]. National Standards for Culturally and Linguistically Appropriate Services in Health Care were designed to reduce disparities in healthcare delivery and to help healthcare systems better address patients’ needs.

Healthcare organizations and providers have a responsibility to reduce racial/ethnic disparities in health to improve equity, and by embedding interventions into their quality improvement initiatives [35]. The Robert Wood Johnson’s Finding Answers: Disparities Research for Change program identified best practices for implementing interventions into healthcare settings to reduce health disparities, including: “1) Recognize disparities and commit to reducing them, 2) Implement a basic quality improvement structure and process, 3) Make equity an integral component of quality improvement efforts, 4) Design the intervention(s), 5) Implement, evaluate, and adjust the intervention(s), 6) Sustain the intervention(s).”

A few culturally competent interventions (e.g., combined home and transplant center education, public awareness campaigns, and patient/family communication with social workers) have been shown to increase LDKT rates [36–42]. Studies also show that increasing knowledge about LDKT increases potential donors’ willingness to donate, living donor inquiries with transplant centers, donor evaluations, and actual LDKTs [39, 43, 44]. Most interventions focus on African Americans [40, 41], [44–46]. Few interventions target Hispanics’ cultural concerns [36] by raising awareness of living donation as a treatment option [21] and increasing willingness to become a living donor via mass media campaigns [21, 42]. Because disparities in minority access to LDKT can be attributed to provider and hospital level factors (e.g., lack of minority healthcare providers, or language or literacy-appropriate educational materials) [47], interventions are needed at the transplant center level. No studies have assessed the effectiveness of culturally competent transplant center-based interventions to increase LDKT in Hispanics. Additionally, none of these interventions have evaluated the implementation of their intervention.

Despite the increasing value placed on improving equity in healthcare organizations, systematic reviews of interventions designed to reduce racial/ethnic disparities in other conditions (e.g., HIV prevention [48], cervical cancer screening, diagnosis, and treatment [49], prostate cancer treatment decision making [50], colorectal care [51], asthma outcomes [52]) found that few interventions entailed culturally targeted components, and that few interventions targeted changing health provider behavior or organizational microsystems [35]. Moreover, few implementation studies designed to reduce ethnic/racial disparities have evaluated the fidelity of their intervention implementation [53, 54].

The implementation science literature has also witnessed a paucity of complex longitudinal interventions at the organizational level that have been evaluated for their implementation [55]. Complex interventions generally involve multiple interacting components, multiple people delivering the intervention, with those delivering the intervention engaging in many and/or difficult behaviors, multiple organizational levels, relatively little flexibility is permitted in tailoring the intervention, and multiple and/or diverse outcomes [56]. Few implementation studies are prospective [57] and even fewer focus on organizational-level interventions [55]. Both complex and multi-year long interventions can make the implementation process more difficult to initiate and sustain, and thereby jeopardize the intervention fidelity and outcomes. Understanding which kinds of complex intervention components are more or less feasible to implement, and how the intervention duration can influence sustainability, are important factors that require further attention.

This protocol describes a theory-driven study to evaluate the effectiveness of the Northwestern Memorial™ Hispanic Kidney Transplant Program as a complex, longitudinal, culturally targeted intervention to increase living kidney donor transplantation among Hispanics, and to ultimately reduce health disparities in kidney transplantation. The specific aims of the study are as follows:

### Specific aims

Aim 1. To implement the HKTP at two transplant centers by conducting a needs assessment of barriers and using a “learning collaborative” model to deliver HKTP protocols, scripts, and materials.

Aim 2. To evaluate how factors from the CFIR and the Revised PARIHS frameworks, and emergent factors unique to the implementation of culturally competent care, affect fidelity.

Aim 3. To conduct a pre-post HKTP intervention evaluation with matched controls to evaluate the effect of the HKTP, and the influence of innovation adaptations, on intermediate and final outcomes.

### Conceptual framework

The revised version of the Promoting Action on Research Implementation in Health Services (Revised PARIHS) [58, 59] and the Consolidated Framework for Implementation Research (CFIR) will be used in both implementation design and testing. Although the CFIR is more comprehensive than any single framework because it comprises nineteen other theories and frameworks [60], it does not include facilitation from the revised PARIHS. From these frameworks, we have identified core concepts that will be used to compare the two transplant centers implementing the HKTP. Successful implementation entails the development of an implementation plan and its realization, the uptake of evidence-based innovation, and the achievement of patient and organizational outcomes [61]. We will examine which of the following factors influence the successful implementation of the HKTP.

**Evidence** focuses on the knowledge of, clinical expertise in, patient preferences for, and relative advantage of the evidence-based practice [59]. The Revised PARIHS framework and the CFIR both argue that settings which have some knowledge of the research supporting the intervention (i.e., cultural differences underlying differential rates of Hispanic LDKT rates), clinical evidence of outcomes (i.e., increased Hispanic LDKT rates), and have patients who show a preference for the intervention will be more likely to successfully implement the intervention.

**Relative advantage** focuses on perceived comparative benefit of the HKTP to current practice for promoting living kidney transplantation. The CFIR argues that sites that perceive the intervention as providing a greater relative advantage will be more likely to implement the protocol with fidelity.

**Complexity** describes the perceived difficulty of implementing the HKTP. The CFIR argues that this perception varies depending on how many processes and practices needed to be reoriented or changed in order to implement the protocol, and as such, varies from context to context.

**Perceptions of the quality & packaging, costs, and adaptability of the protocol** describes several factors from the CFIR that refer to individuals’ perceptions of interventions. These perceptions, irrespective of the objective characteristics of the HKTP, shape willingness to implement the intervention.

**Outer setting** refers to the broader economic, political, and social contexts in the CFIR. Factors from the outer setting, including the degree to which knowledge of patient needs and resources are accurately known, the degree of cosmopolitanism, peer pressure, and external policies and incentives, influence the likelihood of a successful implementation.

**Inner setting** refers to structural, political, and cultural contexts (e.g., communications between transplant staff) through which the implementation process unfolds within the organization [62]. In particular, we examine the role of structural characteristics, culture, implementation climate, and contextual readiness of the transplant center.

**Facilitation** refers to a process of “helping individuals and teams to understand what they need to change and how they need to change it in order to apply evidence to practice.” [58, 63]. The facilitator, or ‘change agent,’ will provide help, guidance, and support to change attitudes, habits, skills, and ways of thinking and working in order to successfully implement the intervention [64]. In the Revised PARIHS framework, facilitation takes a central role. As such, we pay particular attention to how facilitation differences may influence implementation.

**Fig. 1:**
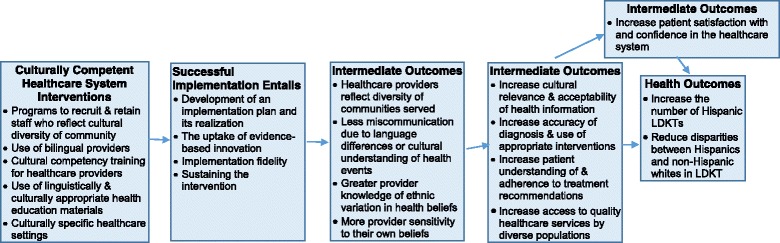
Analytic Framework to Evaluate the Effectiveness of Healthcare System Interventions to Increase Cultural Competence (Adapted from [65])

**The Cultural Competency Analytic Framework** will be used to guide the evaluation of the effectiveness of the HKTP on increasing culturally competent care, as evidenced by the increased number of LDKTs and increased patient satisfaction with care (Fig. 1) [65]. According to the framework, culturally competent healthcare system interventions (e.g., bicultural, bilingual staff) contribute to intermediate outcomes (e.g., less patient-provider miscommunication, increased patient understanding of treatment recommendations, increased patient satisfaction), which leads to improvements in health outcomes (e.g., increased number of LDKTs).

## Methods and design

### Setting

The HKTP will be prospectively evaluated at two intervention sites: Baylor University Medical Center (BUMC) in Dallas, TX, and the Mayo Clinic in Phoenix, AZ. Both sites are located in cities serving the largest U.S. Hispanic (Dallas: 38.3%; Phoenix: 40.8%) and ethnic subgroup (Mexican) populations [66]. Intervention sites are matched with control sites by city population size, Hispanic proportion of the city population, and KT and LDKT volumes, including: Houston Methodist in Houston, TX (matched to BUMC), and University of Colorado Denver in Denver, CO (matched to MC). Control sites were selected in different cities than intervention sites to avoid contamination from local implementation efforts. As Northwestern Memorial Hospital (NMH)/Northwestern Medicine® (NM®) created the HKTP, but did not prospectively evaluate it, NMH/NM serves as the Study Coordinating Center. NMH/NM® is a nationally-recognized, academic teaching hospital, and includes one of the nation’s leading transplant centers.

### Intervention

Northwestern Medicine’s® (NM) Comprehensive Transplant Center launched the Hispanic Kidney Transplant Program (HKTP) in December 2006 to address the needs of the growing underserved Hispanic population in Chicago. The NM® HKTP is culturally targeted by employing bicultural and bilingual staff, using Spanish in patient/family-provider communications and written materials, and addressing commonly shared Hispanic cultural values, beliefs, misconceptions, and information needs of Hispanic ESRD patients and potential living donors [67], as previously described [20, 68]. HKTP’s culturally targeted components correspond closely with the National Quality Forum’s (NQF) Framework for Standardizing Measurement and Reporting of High-Quality, Culturally Competent Care (Table 1) [20, 69].

**Table 1:** Implementation fidelity for each intervention component, and moderating factors affecting fidelity

Intervention component ID #	Intervention component description	NQF dimensions	Data source
*For patients at the dialysis center:*			
1	At the dialysis center, the bilingual (bicultural) outreach staff hold lobby days to introduce, hand out an information folder in Spanish about the HKTP, and invite Hispanic patients to attend the HKTP. Staff provide patient contact information to clinic schedulers to set up a clinic visit for each patient.	Workforce diversity and training; Data collection, public accountability, and quality improvement; Community outreach	-Observations -Checklist -Survey-Metrics
2	Transplant center telephone bilingual* schedulers offer (follow the script) the HKTP clinic as an option to all Hispanic patients calling to schedule transplant evaluation, regardless of the patient’s spoken language. Schedulers encourage patients to bring their family members to the HKTP visit.	Integration into management systems and operations; Workforce diversity and training	-Audio-Recordings
3	Establish a Spanish speaking telephone line at the transplant center.	Integration into management systems and operations	-Survey-Metrics -Learning Collaborative
4	The transplant center’s phone line offers (live) Spanish-speaking schedulers when patients call.	Integration into management systems and operations	-Survey-Metrics
*For patients and family who visit the HKTP:*			
5	Bicultural Transplant surgeon delivers the 2 back-to-back HKTP education sessions that provide same care as the English program as well as address cultural concerns about transplantation in Spanish.	Patient-provider communication; Workforce diversity and training; Integration into management systems and operations	-Observation-Site Visit -Observation-Research Staff -Survey-Metrics -Audio-Recordings
6	Education sessions address Hispanic cultural and religious concerns & myths.	Patient-provider communication	-Audio-Recordings
7	Education sessions include family members in both sessions.	Patient-provider communication	-Observation-Site Visit -Observation-Staff -Survey-Metrics
8	Bicultural transplant surgeon or bilingual transplant nephrologist holds a ‘wrap up’ clinical visit with each patient and asks about available potential living donors.	Patient-provider communication	-Survey-Metrics -Observation-site Visit
9	HKTP bilingual clinical staff and/or outreach staff contact patients who have not completed evaluation within 10–12 weeks.	Care delivery and supporting mechanisms	-Medical chart review
10	HKTP Director calls patients and referring MD if patients have not completed pre-transplant evaluation by 8–10 and 12 weeks.	Leadership; Care delivery and supporting mechanisms; Community outreach	-Medical chart review
11	Availability of interpreters or interpreter services.	Workforce diversity and training	-Phone calls
12	Data collection on patients in a transplant database for quality assurance and identifying areas for improvement.	Data collection, public accountability, and quality improvement	-Phone calls
*For the local nephrology community:*			
13	Letters are mailed to nephrologists introducing the HKTP to increase patient referral.	Community engagement	-Medical chart review
14	Transplant center holds in-service presentations to local social worker and nephrology organizations to increase awareness of the HKTP to increase patient referral.	Community engagement	-Survey-Metrics -Phone calls
*For the public:*			
15	Transplant center engages in marketing events (e.g., interviews in news and social media) about the HKTP directed to the Hispanic community in Spanish (TV, radio, newspaper).	Patient-provider communication; Community engagement	-Survey-Metrics -Phone calls
16	Transplant center website hosts information about the HKTP in Spanish.	Patient-provider communication	-Survey-Metrics -Phone calls

#### Community engagement

Outreach to the community is vital for increasing the number of patients referred to the HKTP. Bilingual/bicultural outreach staff (e.g., social workers) will hold 3 to 4 “lobby days” at dialysis centers per month. This will entail setting up a booth in the lobby to educate dialysis patients in Spanish about transplantation, address questions, and invite patients to come to the HKTP for transplant evaluation. The outreach staff will give patients materials describing the HKTP written in Spanish and English. When patients express interest in transplantation, the outreach staff will obtain patients’ contact information and hand out their business card. Upon returning to the transplant center, the outreach staff will enter a note in the transplant scheduling database and/or call patients back to confirm interest. The note in the database will trigger an email to the scheduler to call the patient to schedule an appointment.

#### Care delivery and supporting mechanisms

Transplant centers will mail letters to all referring nephrology and primary care practice groups to inform them about the HKTP and increase referrals of Hispanic patients. Transplant centers, as part of routine care, notify referring nephrologists by phone or letter when their patients initiate evaluation or receive a transplant; the intervention will incorporate informing nephrologists about the HKTP into such communications. HKTP facilitators and staff will also sponsor in-service education sessions or conferences to educate the local nephrology and social worker communities about the HKTP program. Additionally, transplant center staff and faculty (site PIs) will contact potential recipients, by telephone and/or mailed letter, who have not completed their transplant evaluation to remind them to complete the process.

#### Integration into management systems and operations

When referred Hispanic patients call the transplant center to set up their initial transplant evaluation visit, the schedulers who set up appointments will follow a script. The script will briefly describe the education sessions in the HKTP and the routine English transplant programs, explaining that the HKTP delivers the same medical care as the English program, but is provided in Spanish by bilingual and bicultural transplant clinicians, and addresses Hispanic cultural concerns. At the end of the script, the schedulers will ask Hispanic patients whether they prefer the HKTP or the English clinic. If patients prefer the HKTP clinic, then schedulers will encourage patients to bring friends and family members, including family elders, with them to the HKTP clinic.

#### Patient-provider communication

The Hispanic, bilingual physician (Directors of the HKTP at each site) will deliver HKTP clinics twice per month, each of which entails two sequential educational sessions in a group setting in Spanish, at the beginning of kidney transplant and live donor evaluation. Session 1 will be delivered to potential recipients, family, and potential living donors, and will discuss kidney transplantation, deceased and living donation, and will address cultural and religious concerns commonly held by Hispanics. Session 2 will be delivered to friends, family members, and potential living donors and will cover more specific information about living donation, while potential recipients receive initial clinical evaluation.

Bilingual and bicultural Hispanic transplant clinical staff and faculty will clinically evaluate patients after the education sessions. After Session 1, staff will obtain patients’ history and physical information. After Session 2, the physician Director will hold a wrap-up visit one-on-one with patients and their family members to ask the patient for their verbal report of available living donors who have expressed an interest in donation, and address further questions and misconceptions about donation and transplantation. Potential donors will be evaluated on a separate day to avoid the potential for coercion.

#### Marketing

The intervention will entail marketing to the general public to increase awareness of the HKTP. The transplant centers will revise their current websites by including information about the HKTP in Spanish and English. The site PIs at both transplant centers, who deliver the HKTP education and thus serve as the face of the HKTP, will engage in news media interviews and public service announcements targeting the Hispanic community (e.g., via Spanish-language television, radio, newspaper) about Hispanic disparities in transplantation and the availability of the HKTP to address this problem.

#### Data collection, public accountability, and quality improvement

Both study sites will modify their current transplant databases to account for every Hispanic potential recipient and potential living donor to facilitate patient management and data analysis of patients’ referrals to the HKTP, and their evaluation status until wait-listing and transplant. Databases will also be updated, as needed, to track Hispanic nationality.

### Study design overview

A prospective effectiveness-implementation hybrid design [70] involving pre-post intervention evaluation with matched controls will be used to implement and evaluate the HKTP. Due to design, matched control sites were pre-specified, and were matched with intervention sites based on city population size, Hispanic proportion of the city population, and kidney transplant and LDKT volumes. Although an ideal study design would be a randomized controlled trial that randomizes patients within each center, it is laden by contamination effects. Randomizing at the center level would require a large number of centers to serve as intervention sites and controls, which is costly. To assess other secular trends, data from matched control centers that do not implement the HKTP will be compared. Control sites were selected in different cities than intervention sites to avoid contamination from local implementation efforts. The first year will focus on implementing the HKTP at BUMC and MC over a 12-month period (Aim 1). Remaining years will focus on evaluating the HKTP on Hispanic LDKTs and on program fidelity in years 2–4½ (Aims 2 and 3).

The hypotheses are that the HKTP will lead to a(n):

- H_1_ Increase in the ratio of Hispanic to non-Hispanic white LDKTs and in the number of Hispanic LDKTs
- H_2_ Increase in the number of Hispanic ESRD patients added to the waiting list
- H_3_ Increase in the number of Hispanic potential donors per potential recipient
- H_4_ Greater satisfaction of Hispanic ESRD patients with culturally competent care

AIM 1: To implement the HKTP at Two Transplant Centers.

The NU team will first provide BUMC and MC with a preliminary set of protocols comprising the HKTP manual of operations, scripts for clinical coordinator and outreach staff to use, and educational materials describing the structure and content of the education sessions and transcriptions. To train the staff and providers at BUMC and MC, a video-based curriculum will be provided that explains the outreach staff’s communications with dialysis patients, and demonstrates NM’s HKTP Director’s education sessions. NM’s HKTP education session PowerPoint slides will also be provided for sites to use. The set of protocols will help BUMC and MC to determine how to modify their organizations to accommodate the HKTP. Publications that provide evidence to support the HKTP’s increase in Hispanic LDKTs will be provided to foster site ‘buy in’ to implement the HKTP [20, 68].

*Site Visit* Dr. Gordon and Dr. Caicedo (co-PIs) will conduct a site visit in year 1 to perform the needs assessment to identify transplant provider-, administrator-, and center-related barriers and facilitators to the implementation and sustainability of the HKTP, in order to refine and deliver the final set of protocols, scripts, and educational materials. During the needs assessment, Dr. Gordon will use ethnographic methods [71] to interview, observe, and survey BUMC’s and MC’s transplant physician (and/or site-PI), staff (outreach staff), and administrators involved in HKTP implementation (total n = ~ 5 per site).

*In-Depth, Qualitative Interviews* will assess the current workflow and where changes could be made within the workflow to comply with the HKTP protocol. Interviews will be audio-recorded and last 30–45 min.

*The “Learning Collaborative”* [72, 73] method will be used to help BUMC and MC staff design center-customized approaches and solutions to barriers to implementing the HKTP. This short, rapid approach to healthcare quality improvement is often used by organizations and providers to work collaboratively by sharing their experiences and best practices to accelerate learning [74, 75]. This approach can increase critical thinking and leadership skills more than individual learning [76, 77], and enhance the observability and trialability of the HKTP -- characteristics identified as important in Revised PARIHS evidence framework. Staff from BUMC and MC will visit the NU research team in month 7 for a 2-day “learn together” program [78] to discuss their challenges and strategize on overcoming problems with implementing the HKTP at their institutions. During the in-person meeting, BUMC and MC staff will shadow and role-play with NU staff to practice communicating with patients and responding to patients’ questions. Additionally, the Learning Collaborative will entail routine telephone calls between program staff to benchmark against each other and NM’s HKTP to identify best practices and learn from one another. The NU research team will continuously help troubleshoot ways that BUMC and MC can increase readiness and adapt the HKTP into their programs, using NQF guidelines and the cultural competence analytic framework [65, 69]. Through collaborative learning, BUMC and MC staff will obtain the social support necessary to overcome isolation of going through the implementation process alone.

Based on data analysis of the needs assessment, a subsequent, final set of protocols, scripts, and materials that integrate modifications to the HKTP that adapt the HKTP to each intervention site will be created and provided. Remediation plans will be used to assist intervention sites in implementing the protocols, as needed, if deficiencies are detected by the research staff or observed during site visits.

Formative evaluation procedures will be used to optimize intervention implementation (fidelity) [79], and enhance the HKTP, as the NIDDK program announcement requires [80]. Specifically, the delivery of *dosage* and *coverage* of required programmatic elements will be evaluated to determine whether all intervention components were implemented as planned, and to identify which parts of the HKTP worked best. Monitoring will occur weekly during implementation (months 0–8), twice per month for adjustment (months 9–12), and monthly for process checks (months 13–54). Given that people’s actions typically differ from what they say they do [71], we will use mixed methods [81] to assess actual behaviors.

AIM 2: To evaluate how factors from the CFIR and the Revised PARIHS frameworks and emergent factors unique to the implementation of culturally competent care affect fidelity.

### Mixed methods research

We will use a mixed-methods approach using qualitative and quantitative research methods to obtain broader insights than either method could provide alone, to enable further clarification of findings generated by a single method, and to enhance the validity of findings [81–83]. Qualitative methods include: site visits, semi-structured interviews, observations, and routine telephone discussions. Quantitative methods will include surveys and checklist evaluations.

*Routine telephone calls* will be conducted by Dr. Gordon with a NU research staff member, and each site’s research staff member on a weekly basis for the first 18 months, and on a biweekly basis thereafter. During the phone calls, Dr. Gordon will informally ask about the progress of the IRB, the delivery of HKTP clinics, number of patients, barriers to implementation, problems with delivery, and brainstorm solutions. The phone calls will last 30–60 min. Dr. Gordon will also hold ad hoc telephone meetings or email correspondences with each physician (site PI) for updates and progress made on the implementation and intervention delivery. Field notes will be taken during all correspondences.

*Learning collaborative telephone calls* will be conducted quarterly by Dr. Gordon with a NU research staff member and appropriate front-line staff from the two transplant centers. These phone calls will last approximately 60 min. During these phone calls, comparative intermediate outcomes will be benchmarked from the two transplant centers, and staff will share best practices they have developed for overcoming obstacles and achieving better results. These interviews will be recorded and transcribed.

*Routine site visits* will be conducted by Dr. Gordon and Dr. Caicedo each year to assess progress, extent of tailoring, strengths, and obstacles related to the HKTP implementation.

*Semi-structured Interviews* will assess organizational readiness to change, each site’s disposition to using evidence for adapting the HKTP into practice, organizational culture, and barriers and facilitators to implementing each respondent’s respective HKTP component, using Stetler and colleagues’ Guide for each revised PARIHS construct [58]. Interviews will be conducted in person (during site visits) or by telephone with the clinical team (e.g., faculty, administrators, staff) and research staff, will last 30–60 min, and will be audio-recorded and transcribed.

*Organization surveys* will assess BUMC’s and MC’s readiness to change, facilitation capacity, and disposition to accepting evidence about the HKTP’s effectiveness to implement change, using the validated, Organizational Readiness to Change (ORCA) instrument (Cronbach’s alpha = 0.74–0.95) [84]. ORCA surveys will be self-administered on paper by the clinical team and research staff before the first site visit.

*Online surveys* will be completed by the research staff (via Research Electronic Data Capture (REDCap)) that assess metrics about key HKTP components on a monthly, quarterly, or biannual basis based on observation or information provided by the outreach staff. For example, the frequency of outreach visits to dialysis centers per month, the number of Hispanic patients met per visit, the number of patients who express an interest in coming to the HKTP for evaluation per visit, the number of these patients who share their contact information with the outreach staff, the number of these patients who schedule an appointment, the number of patients who show up to each HKTP education session, the number who complete the evaluation process and get waitlisted for transplantation, and the number who get a transplant by type of transplant. Additionally, the research staff member will track through online surveys the number of HKTP education sessions per month, and marketing efforts. Finally, research staff will assess whether their hospital’s patient advocacy department has received any concerns from patients or families with the HKTP program on a quarterly basis through a single-item survey.

*Observations* will be conducted in two ways. (1) During the site visits, Dr. Gordon will conduct observations of the dialysis outreach, telephone communications, and educational sessions in practice to see how they occur and to learn about the organizational culture. (2) During routine intervals, research staff will shadow outreach staff as they perform outreach at dialysis centers, and observe faculty delivering the education sessions monthly (months 13–54) to identify potential discrepancies from the HKTP protocols and scripts to amend.

*Checklists* will be completed by research staff while observing the outreach staff and the HKTP education sessions to track adherence to HKTP protocols and scripts. Completed checklists, metrics, surveys, and audio-recordings will be reviewed by the NU research team to identify potential problems and solutions to improving the HKTP on a quarterly basis.

*Audio-Recordings* will be made of two HKTP components: (1) telephone conversations between schedulers and patients calling to schedule an appointment, one day per month. Patients (*N* = ~ 75) will first be informed that “This call may be monitored for quality control” and asked for their verbal consent to be audio-recorded. (2) HKTP educational sessions led by the site PI delivered to ESRD patients and their family, once per month. All recordings will be electronically sent to the NU research team. Inter-rater reliability will be assessed by the intervention site’s research staff and NU’s research staff [85] to identify gaps or errors in the delivery of telephone communication or educational content, and determine whether retraining is needed.

### Qualitative analysis

Qualitative data and field notes from the needs assessment and follow-up interviews will be analyzed for themes and patterns emergent from the data [86, 87], using the constant comparative [88], deductive and inductive coding methods [89]. Drs. Gordon and Shumate will develop an initial deductive code list a priori, based on the PARIHS and CFIR framework domains, and Dr. Gordon will train the research staff on how to apply it to interview transcripts and field notes. Analytic retreats will then be held with research staff (led by Drs. Gordon, Shumate) to inductively develop additional codes within each domain, in an iterative process. The retreats will entail comparing codes, resolving discrepancies in code meaning and use through discussion, and revising the codebook. Codes will be revised to account for new observations, continuing until saturation is reached [90]. Analytic insights and decisions made during the retreats will be captured in minutes taken by the research staff. Qualitative analysis software (MaxQDA) will be used to support analysis, and Dr. Gordon will meet with the research staff to monitor coding quality and timeliness.

We will use Stirman, Miller, Toder and Calloway’s framework for coding modifications [91] and classification system of reasons for adaptations to evidence-based interventions [92]. This coding scheme will allow us to assess the types of content and contextual modification to the HKTP. Additionally, through qualitative analysis, we will determine the reasons for these modifications. Finally, we will determine the impact of these modifications on the outcomes of the HKTP from outreach to transplant. We will compare similarities and differences in modifications across study sites.

### Statistical analysis

Descriptive statistics of metrics will be used to assess extent of fidelity to implementation. Bivariate analyses involving t-test comparisons of ORCA data will be conducted to compare level of readiness between study sites.

AIM 3. To conduct a pre-post HKTP intervention evaluation with matched controls to evaluate the effect of the HKTP, and the influence of innovation adaptations, on intermediate and final outcomes.

### Outcome measures

We will assess the reduction in disparities between self-identified Hispanics and non-Hispanic whites in LDKT rates for adult-to-adult living kidney donation. The effectiveness of the HKTP will be assessed by comparing the outcomes before introducing the HKTP and after the HKTP has been fully implemented. Measures will be collected at intervention and control sites in pre and post periods. For the final outcome measures, de-identified, aggregate center-level data will be collected from the Organ Procurement and Transplant Network/United Network for Organ Sharing (OPTN/UNOS) as publically reported data (Table 2). Data from sites’ medical record review will also be collected. Intermediate outcome measures about each Hispanic and non-Hispanic white potential recipient and potential donor will be collected monthly by research directly from intervention and control sites by medical record review to assess the pathways by which the HKTP potentially increases rates of Hispanic LDKT. We expect that the pre-post change will demonstrate a higher number of potential donors who initiate evaluation and complete more steps in the donor evaluation process.

**Table 2:** Final and Intermediate outcome measures: transplant center-reported

Construct	Measure description
Final outcomes	
Ratio of Hispanics to Non-Hispanic Whites Receiving LDKTs	This ratio takes into account growth in transplant center volume while also providing a comparison to non-Hispanic whites, necessary for assessing disparities in LDKT [102]. The ratio provides an index of whether the Hispanic LDKT increase has grown over and above the increase observed at a center overall. By using this ratio, factors related to the center’s infrastructure and capacity to perform additional LDKTs that affect both Hispanics and non-Hispanic whites equally are accounted for. Institutional commitments to LDKT that are associated with higher rates of LDKT, can influence willingness to donate or preferences for LDKT, and affect both populations equally (e.g., paired donation, and desensitization efforts), are accounted for.
Number of Hispanic LDKTs	Change in number of Hispanic LDKTs compared to change in number of non-Hispanic white LDKTs will provide evidence that the increase is not at the expense of white patients.
Number of Hispanic Patients Added to the Waiting list:	An increase in this number indicates success in overcoming barriers in gaining access (referral) to transplantation and completing the evaluation [103].
Number of Hispanic Potential Donors per Potential Recipient	An increase in this proportion reflects success in encouraging more potential living donors to undergo donor evaluation.
Intermediate Outcomes	
# Patients referred by outreach	The aggregate number of patients who were referred by outreach staff. Referral can be made by self, staff at dialysis centers, nephrologists, primary physicians, or transplant outreach staff.
# Patients scheduled/attending the education session	The aggregate number of patients who were scheduled and attended the HKTP education sessions.
# Patients who completed evaluation	The aggregate number of patients who completed the transplant evaluation process.
# Patients waitlisted	The number of additions to the transplant center’s own waiting list will serve as a proxy for access to the waiting list.
# Days on waiting list, dialysis time	The number of days patients have waited on the waiting list to date.
# Pre-emptive transplants	The number of patients who received a living donor transplant prior to going on dialysis.
# Potential donors at each phase of evaluation	The aggregate number of potential living donors who have completed each phase of the donor evaluation process.
# Days for donor to complete each phase of evaluation	The number of days from start to finish of each phase of living donor evaluation from initiation to clearance for donation to donation.
Potential donor disposition	The donor’s evaluation status as: initiated, completed, ruled out, withdrew, alternate donor found.
Contextual Outcomes	
Patient and potential donor socioeconomic status	Age, gender, race/ethnicity, insurance.
Organ Procurement Organization and transplant center factors	ESRD incidence, and transplant center volume.

Hispanic patients’ and family members’ satisfaction with culturally competent care will be evaluated before and after implementing the HKTP by using the validated, closed-ended, DHHS AHRQ Consumer Assessment of Healthcare Providers and Systems (CAHPs) Cultural Competence Item Set (34 items) [93, 94] and Patient Centered Medical Home subset (8 items) [88, 89]. Topics covered include: patient-provider communication, shared decision making, preferences for shared decision making, experiences leading to trust or distrust, experiences of discrimination, and linguistic competency. Surveys will be self-administered on paper in the clinic waiting room after completing clinic visits that day. A total of *n* = 70 patients/donors will be surveyed at each site in year 1 (pre-HKTP) and year 5 (post-HKTP) [93], in English or Spanish [95].

Contextual measures will also be collected from all sites to control for confounders through routine phone call communications with study sites. News items and policy changes will also be scanned for secular effects on data collection or HKTP outcomes. For example, the research team will take note of international, national, regional, and hospital news items pertaining to health disparities, Hispanics, and/or organ transplantation/donation.

### Statistical analysis plan

SAS version 9.4 (Carey, NC) will be used for analyses. Testing of each hypothesis will be performed separately for each study site. For Hypotheses 1 and 2, quarterly UNOS outcome data (6 years pre- and 3.5 years post-HKTP) will be used to model ethnic and racial differences in transplants, to account for external events that could alter LDKT rates, and to analyze the sustainability of the HKTP intervention. Hypotheses 3 and 4 will be evaluated with monthly data. Associations between final outcome and intermediate outcome measures, and individual patient and donor level variables will be evaluated. Matched intervention and control sites will be compared.

To test hypothesis 1, we will use OPTN/UNOS data, to construct the ratio of the number of Hispanics receiving LDKTs to the number of non-Hispanic whites receiving LDKTs for each center from 6 years prior to 3.5 years after implementation of the HKTP. An interrupted time series (ITS) design with segmented regression analysis will be used to assess the HKTP. Ratio data will be collected across 24 quarterly time points pre-intervention (reference point), and 14 time periods post-intervention for a total of 38 time-series data points for each center.

A probabilistic sensitivity analysis will be conducted to ascertain the likelihood that changes in the volume of LDKTs at study sites are indeed attributable to implementation of the HKTP. Rather than assigning a single value to each parameter, we will assign a distribution to all parameters in the model. The ranges will be determined by the average value, the standard deviation, and the shape of the spread of the data. A 1-way sensitivity analysis by varying one parameter at one time will be used to identify secular trend threshold values. We will collect intermediate outcome data at control sites to enable more nuanced pretest-posttest analyses. Sensitivity analyses using published [96, 97] and publically reported LDKT volume data from matched controls will be used to adjust for regional variability. In addition, the two transplant centers implementing the HKTP will be compared to each other. To assess the influence of innovative adaptations on outcomes, qualitative data from routine assessments of the modifications to the HKTP (Aim 2), collected at 14 quarterly time periods post-intervention, will be transformed into quantitative parameters, and will be incorporated into a longitudinal data analysis evaluating trend in outcomes over the post-intervention period accounting for quarterly time-varying adaptations.

We expect an increase in satisfaction scores before and after the education sessions by 10% in post-HKTP based on prior studies [98]. Differences in CAHPS composite scores will then be compared between pre- and post-HKTP by each site using a two-sample t-test. A total of 100 patients per site (50 pre-HKTP, 50 post-HKTP) provides 80% power to detect a difference of 10% (SD = 18%) in mean satisfaction scores before and after education sessions between pre- and post-HKTP groups using a two-sided t-test with a *p* = 0.05 significance level.

## Discussion

This research has the potential to make several important contributions to implementation science. First, the HKTP is a complex intervention targeted at the organization-level. Most implementation science research on changing healthcare professionals’ behavior has focused on changing an individual’s behavior rather than changing a healthcare organization [55]. As such, this research will uniquely examine the degree to which the CFIR and revised PARIHS frameworks describe the factors that influence the implementation of this type of intervention.

Second, this research examines the long-term implementation of the HKTP, with five years of observations. As such, it contributes to sparse research on how organizations sustain complex interventions over time [99].

Third, this research uniquely examines the implementation of an intervention focusing on reducing healthcare disparities. As such, it has the potential to expand the factors described in the CFIR framework. Specifically, this research will draw attention to how healthcare professionals’ perceptions of different patient groups may impact the implementation of the protocol.

Finally, this research uses an effectiveness-implementation hybrid design to examine both the antecedents to implementation fidelity and its outcomes [70]. In particular, this research is poised to explain both the factors that led to particular types of modifications to the design and the impact of those modifications on patient outcomes [91].

This study will advance knowledge of implementation science of culturally targeted interventions. Given that Hispanics comprise the largest and fastest growing minority population in the U.S. [14, 15], and are at disproportionately higher risk of ESRD requiring transplantation, [100] this study is expected to have a substantial and sustained impact on reducing health disparities in Hispanics. To our knowledge, the HKTP is the only culturally competent transplant program in the U.S. that upholds National Standards for Culturally and Linguistically Appropriate Services [101]. By implementing and evaluating the HKTP at other transplant centers, we expect to significantly increase LDKTs in Hispanics, and thereby save lives and improve the health of patients by leaving dialysis sooner and increasing pre-emptive KT. As participating transplant centers are located in two different geographic regions, the generalizability of study findings will be enhanced.

We recognize some study limitations and identified ways to overcome those threats. First, the 3.5 year study period may yield conservative estimates; however, at NM®, most transplant candidates who receive a LDKT, do so between 6 and 18 months after completing evaluation. Second, there may be “spill over” of HKTP benefits to non-Hispanic whites. While favorable to society, spill over would support the null hypothesis. However, using number of Hispanic LDKTs as an outcome measure will control for this prospect. Third, it may be challenging to identify which HKTP components are most effective in contributing to the outcomes. However, we will map intervention components to intermediate outcomes, and map intermediate outcomes to final outcomes. Fourth, if we find a lack of significant increase in LDKT rates, then fidelity evaluation, and patient- (demographic) and national-level factors (i.e., media publicity about LDKT) will be assessed for broader LDKT trends.

## Conclusions

We expect our study to contribute to the understanding of complex interventions and hybrid designs in several regards. The HKTP is unlike other interventions evaluated through implementation science because it comprises a complex intervention involving multiple components directed at the micro-organizational level that will be examined over several years. As the intervention is designed to enhance equity in health outcomes, we expect to advance the literature on the challenges of implementing culturally competent care.

## Abbreviations

BUMC: Baylor University Medical Center
CAHPS: Consumer Assessment of Healthcare Providers and Systems
CFIR: The Consolidated Framework for Implementation Research (CFIR)
ESRD: End-stage renal disease
HKTP: Hispanic Kidney Transplant Program
ITS: Interrupted time series
LDKT: Living donor kidney transplantation
LKD: Living kidney donation
MC: Mayo Clinic, Phoenix
NM: Northwestern Medicine
NMH: Northwestern Memorial Hospital
NQF: National Quality Forum
OPTN/UNOS: Organ Procurement and Transplant Network/United Network for Organ Sharing
ORCA: Organizational Readiness to Change
PARIHS: Promoting Action on Research Implementation in Health Services
REDCap: Research Electronic Data Capture
US: United States
